# Comparative accuracy of the REBA MTB MDR and Hain MTBDR*plus* line probe assays for the detection of multidrug-resistant tuberculosis: A multicenter, non-inferiority study

**DOI:** 10.1371/journal.pone.0173804

**Published:** 2017-03-24

**Authors:** Joshua Havumaki, Doris Hillemann, Nazir Ismail, Shaheed Vally Omar, Sophia B. Georghiou, Samuel G. Schumacher, Catharina Boehme, Claudia M. Denkinger

**Affiliations:** 1 Foundation for Innovative New Diagnostics, Geneva, Switzerland; 2 National Reference Laboratory for Mycobacteria, Forschungszentrum Borstel, Borstel, Germany; 3 Centre for Tuberculosis, National Institute for Communicable Diseases, Johannesburg, South Africa; National Institute for Infectious Diseases (L. Spallanzani), ITALY

## Abstract

**Introduction:**

Despite recent diagnostic advances, the majority of multidrug-resistant tuberculosis (MDR-TB) cases remain undiagnosed. Line probes assays (LiPAs) hold great promise to curb the spread of MDR-TB as they can rapidly detect MDR-TB even when laboratory infrastructure is limited, yet few of these assays are currently widely available or supported by World Health Organization (WHO) policy.

**Methods:**

The aim of this prospective, blinded, non-inferiority study was to compare the performance of YD Diagnostics REBA MTB MDR LiPA (YD) to the WHO-endorsed Hain MTBDR*plus* V1 LiPA (Hain V1) for the detection of rifampicin and isoniazid resistance. In phase 1, YD and Hain V1 diagnostic performance was assessed with selected culture isolates and results were compared to phenotypic drug susceptibility testing (DST) results and targeted sequencing data. In phase 2, both assays were tested on processed sputum samples and results were compared to phenotypic DST results.

**Results:**

In phase 1, YD did not achieve non-inferiority to Hain V1. For isoniazid resistance detection, Hain V1 had a sensitivity of 89% (95%CI 83.8–93%) and specificity of 99.4% (95%CI 96.9–100%). While YD had a similar sensitivity of 92% (95%CI 87.3–95.4%), the specificity was inferior at 92.6% (95%CI 87.6–96%). For rifampicin resistance detection, Hain V1 had a sensitivity of 90.2% (95%CI 84.8–94.2%) and specificity of 98.5% (95%CI 95.7–99.7%) while YD had an inferior sensitivity of 72.4% (95%CI 65.1–78.9%) and a comparable specificity of 98% (95%CI 95–99.5%). Similar results were observed in phase 2. For MDR-TB detection, the sensitivity and specificity of Hain V1 was 93.4% (95%CI 88.2–96.2%) and 96.2% (95%CI 88.2–96.8%), respectively, compared to 75.7% (95%CI 68–82.2%) and 92% (95%CI 88.2–94.9%) for YD.

**Conclusions:**

YD did not achieve non-inferiority with Hain V1. Further improvements and repeat evaluation of YD is necessary prior to recommending its use for clinical settings.

## Introduction

Despite advances in the diagnosis of multidrug-resistant tuberculosis (MDR-TB), i.e. TB that is resistant to the standard first-line drugs rifampicin (RIF) and isoniazid (INH), MDR-TB diagnostic coverage remains insufficient. In 2015, only 20% of the estimated 580,000 MDR-TB cases were diagnosed globally, and MDR-TB prevalence rates have remained unchanged in recent years [1, 2].

Molecular MDR-TB diagnostics hold great promise as rapid, sensitive and cost-effective tools to curb the spread of MDR-TB. These tests are based upon the identification of genetic mutations in MTB that confer resistance to RIF and INH; more than 95% of all RIF-resistant isolates have key mutations in the 81 base pair resistance-determining region of *rpoB* [3], while INH resistance has been associated with mutations occurring in three different gene regions (the *katG* codon 315 region occur in approximately 66% of resistant strains, mutations in the *inhA* promotor occur in 21% of resistant strains, and mutations in the *oxyR-ahpC* intergenic region occur in 5% of resistant strains, globally) [4]. MDR-TB line probe assays (LiPAs) are molecular diagnostic assays that detect drug resistance based upon the hybridization of these specific mutations to complementary probes. The MTBDR*plus* V1 LiPA (Hain V1) was developed in 2008 to detect resistance-associated mutations in *rpoB*, *katG* and the *inhA* promoter. The assay demonstrated excellent performance in field trials, with pooled sensitivity and specificity estimates of 98.1% and 98.7%, respectively, for RIF resistance detection across patient subgroups [5], and was subsequently endorsed by the WHO for clinical detection of RIF resistance [1, 5]. In 2011, two new LiPAs (an updated Hain assay (Hain V2) and the Nipro NTM+MDR-TB Detection Kit 2) covering the same gene regions as Hain V1 were found to be non-inferior to Hain V1 for MDR-TB detection [6], and also received WHO-endorsement in 2016 [1].

Recently, YD Diagnostics (Gyeonggi-do, Republic of Korea) developed a new LiPA, the REBA MTB MDR LiPA (YD), which has expanded coverage for INH resistance detection with its ability to detect mutations in the *oxyR-ahpC* intergenic region. The new LiPA demonstrated promising results in preliminary trials, with one study showing high overall sensitivity and specificity (98.4% and 100%, respectively) for MDR-TB detection compared to sequencing data [7]. An additional evaluation of YD with clinical isolates found improved sensitivity for MDR-TB detection, from 73.1% to 79.9%, due to the addition of the *ahpC* promoter mutations for INH resistance detection [8]. Although these initial results from industry-sponsored studies are promising, additional independent studies are needed to confirm whether the expanded YD assay coverage increases assay sensitivity for MDR-TB detection and to evaluate YD non-inferiority against the WHO-endorsed Hain V1 LiPA. In this study, we compare the performance of the YD LiPA to the Hain V1 LiPA in MTB clinical isolates and sputum specimens in a multisite, multi-country study performed independently from the manufacturer.

## Materials and methods

### Study overview

This multicenter non-inferiority study was conducted between March 2015 and January 2016. The study was conducted in Forschungszentrum Borstel, the National Reference Center for Mycobacteria, in Germany and The Centre for Tuberculosis, National Institute for Communicable Diseases, in Johannesburg, South Africa. The study aim was to compare the diagnostic performance of the index test (YD) to Hain V1, with phenotypic DST and targeted sequencing serving as the reference standard. This study was part of a larger, non-inferiority study that resulted in WHO endorsement of the Nipro and Hain V2 LiPAs [1, 6]. Specific results for the YD assay have not been reported previously.

The study consisted of two phases, as described previously [6]. Briefly, in phase 1, each site was sent a different set of 200 randomly-selected clinical isolates from the Research and Training in Tropical Diseases and Institute for Tropical Medicine (ITM) collections that had been previously characterized by phenotypic DST and DNA sequencing [6]. Each strain set comprised approximately 100 RIF-resistant/INH-resistant, 10 RIF-susceptible/INH-resistant, and 80 RIF-susceptible/INH-susceptible MTB complex strains to assess assay performance, and 10 previously-characterized non-MTB complex (NTM) strains to assess assay specificity for MTB. The sites were blinded to all information for the selected strains [6]. Strains were tested by YD and Hain V1 and results were compared to a composite reference standard of phenotypic DST and available sequencing results. In phase 2, remnant, post-processed sputum sample sediments were consecutively selected for LiPA testing. De-identified samples from 274 patients from Azerbaijan and Moldova were tested in Germany, while samples from 214 locally-recruited patients from the National Health Laboratory Service were tested in South Africa. All samples were tested by both LiPAs and compared to phenotypic DST results.

### Phase 1 sample processing

First-line mycobacterial indicator growth tube (MGIT) 960 DST was conducted for all selected TB strains prior to this study [6]. WHO standard critical concentrations of 1 μg/ml and 0.1 μg/ml were used to define phenotypic resistance to RIF and INH, respectively [9]. Targeted Sanger sequencing of the resistance-associated gene regions of the *katG*, *inhA* and *rpoB* genes was also performed prior to this study [6]. Sequencing results were recorded for the gene regions covered by the LiPAs. After genotypic and phenotypic characterization, the strains were stored in phosphate buffer or molecular grade water (PH 7.0) at -20° or -80°C until the time of this study. Both the index test (YD), and the comparator test (Hain V1) were performed on each sample according to the manufacturers’ instructions [7, 10]. Each LiPA assay used 5μl of the same DNA suspension.

### Phase 2 sample processing

For phase 2, decontaminated sputum sample sediments were suspended in 1.6 to 2.0 ml sterile phosphate buffer (pH 7.0) [6]. Samples were characterized by Ziehl-Neelsen or Auramine microscopy and inoculated into solid and/or liquid media for TB growth detection. Culture positive samples were speciated and phenotypic DST was conducted on all MTB complex samples by MGIT SIRE at the respective sites. All leftover sediments of the decontaminated sputum specimens were stored at -20°C or -80°C and later thawed for LiPA testing, as previously described [6]. 500 μl of each sample was inactivated and DNA was isolated by heating the cells at 100°C on a heat block [6]. Extracted DNA was tested by both Hain V1 and YD, as below.

### Line probe assays

A negative control (water) was included in each LiPA run. As per manufacturers’ instructions [7, 10], test results were regarded as ‘invalid’ if the conjugate/color control, amplification control, marker lane or any loci control was not developed, or if the positive control was negative. In the phase 2 study, the absence of the *katG* loci control was not regarded as ‘invalid’ unless a valid *katG* result was obtained by Hain V1 for the same sample, or vice versa, as certain clinical TB strains have been identified that lack the *katG* gene [11]. Test results were recorded as ‘indeterminate’ if all controls were valid, but the banding pattern did not produce a conclusive result regarding drug resistance. If an assay result was invalid or indeterminate in this study, the sites were instructed to repeat the LiPA once for the given sample. If MGIT phenotypic DST results were contaminated or missing, the sample was excluded from analysis. Invalid, indeterminate and contamination rates are reported separately in this analysis. Performance accuracy calculations are based on the repeat results of invalid or indeterminate initial runs.

### Data analysis

Initially, the sensitivity and specificity of the assays for RIF, INH and MDR-TB detection were computed along with confidence intervals (CI) using the exact binomial method (Clopper-Pearson confidence intervals) [12]. A composite reference standard was used in phase 1 (S1 Table), combining phenotypic DST and targeted Sanger sequencing results, while only phenotypic DST was used as the reference standard in phase 2. The non-inferiority comparison was based on sensitivity and specificity differences between the LiPAs (YD minus Hain V1). Confidence intervals for differences in proportions were computed using Tango’s CI [13], to account for the paired nature of the data. If the lower limit of the difference in sensitivity/specificity was above the non-inferiority margin, YD was considered non-inferior for that parameter [14, 15]. All comparisons were illustrated by forest plots. Non-inferiority margins were set a priori (Table 1), by comparing the pooled performance of MTBDR*plus* V1 from the Ling et al. meta-analyses [5, 16].

**Table 1:** Non-inferiority margins for each accuracy comparison between the YD and Hain V1 line probe assays.

	RIF		INH	
	Sensitivity	Specificity	Sensitivity	Specificity
Non-inferiority margin using composite reference standard (phase 1)	-3%	-2%	-10%	-5%
Non-inferiority margin using phenotypic reference standard (phase 2)	-5%	-4%	-12%	-7%

Non-inferiority margins for each accuracy comparison between YD and Hain V1.

A per-probe, sub-analysis of YD phase 1 results was also conducted using Sanger sequencing results from the same set of strains, to determine whether YD LiPA performance lapses were linked to the performance of specific probes. Diagnostic performance of probes that bound to the *rpoB*, *inhA* and *katG* gene regions were assessed individually. Coverage of each probe was compared to sequencing results to assess assay performance by probe. For instance, a true positive wild type probe is when a probe is absent at a site with a non-silent mutation.

All statistical analyses were performed using R version 0.99.896. YD results for NTM are included in the supporting information. YD performance comparisons to Hain V1, as well as comparisons to Hain V2, are also included in the Supporting Information (S2 and S3 Tables and S1 and S2 Figs).

### Study ethics

Informed consent was waived for this study, as only remnant, de-identified, decontaminated sputum samples were used, no investigational device test results were provided to the healthcare provider or study participants during the study, and all data were analyzed anonymously. Research ethics committee approval was obtained from the Human Research Ethics Committees of University of Witwatersrand in Johannesburg, South Africa, and the Ethics Committee of the University of Lübeck in Germany. The trial (NCT02984579) is registered at ClinicalTrials.org.

## Results

### Indeterminate results

In phase 1, 0.5% (2/400) of MTBC strains were indeterminate by YD for both RIF and INH resistance detection. Two additional strains (0.5%) were indeterminate by Hain V1 for both RIF and INH resistance detection. Of the 20 NTM strains tested, only eight (20%) were correctly identified by YD. Additional phase 1 NTM results are given in the supplement (S1 Text). During phase 2, 1.4% (6/440) sputa were indeterminate for INH and/or RIF resistance detection by YD: one was INH indeterminate, one was RIF indeterminate, and four were both INH and RIF indeterminate. All six MTBC samples were tested in the South African reference laboratory. No Hain V1 samples were indeterminate in phase 2.

### Phase 1 results

Altogether, 400 previously-characterized strains were tested by the YD and Hain V1 assays. Twenty-one total strains were NTM and were excluded from analysis, while four additional strains either did not yield results one or both LiPAs (Table 2), leaving 375 strains for the comparative accuracy analysis.

**Table 2:** Phase 1 sample characteristics and total number of samples included in the line probe assay comparative performance assessment by site.

	South Africa	Germany	Total
Total sent to site	200	200	400
NTM	10	11	21
No Hain V1 test results	1	1	2
No YD results	1	0	1
Indeterminate YD results*	1	0	1
Total excluded	13	12	25
**Total analyzed**	187	188	375

NTM, nontuberculous mycobacteria. Exclusions by site for strain data.

*The indeterminate YD result was based upon repeat.

**Fig 1:**
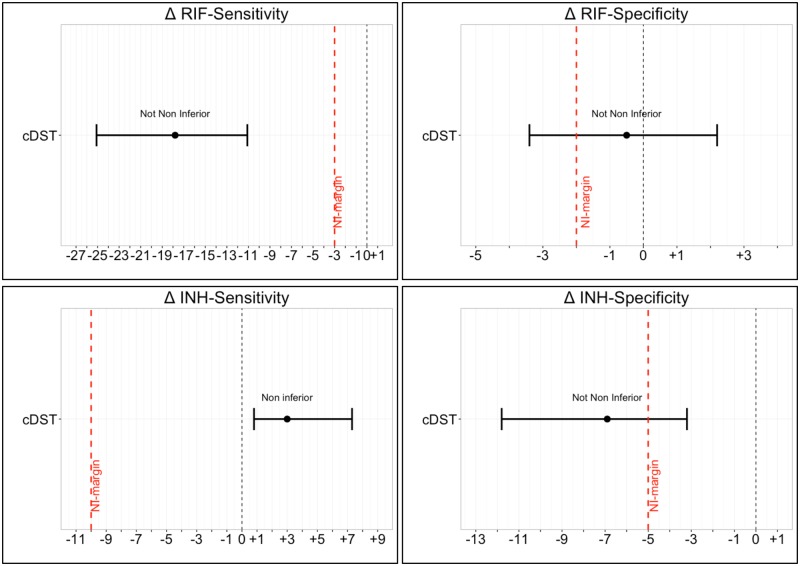
Phase 1 comparative accuracy of the YD line probe assay versus Hain V1 line probe assay on characterized strains. The difference in sensitivity/specificity (Δ = YD–Hain V1) is displayed together with the CIs for the difference in each plot. The horizontal axis indicates the percentage difference between tests. The point in the center of each CI represents the point estimate and whiskers representing the upper and lower limit of the 95% CIs. The black vertical dotted line (where visible) indicates zero difference in sensitivity/specificity and the red vertical broken line indicates the non-inferiority margin. Non-inferiority is demonstrated for a given comparison if the lower limit of the 95%CI does not cross the non-inferiority margin.

Non-inferiority of the YD assay, compared to the Hain V1 assay, was only achieved for sensitivity for INH resistance detection: Hain V1 had a sensitivity of 89% (95%CI 83.8–93%), while YD had a sensitivity of 92% (95%CI 87.3–95.4%) (Table 3). Non-inferiority was not met for all other comparisons. The sensitivity of the assays for RIF resistance detection showed the largest discordance: 90.2% (95%CI 84.8–94.2%) for Hain V1 and 72.4% (95%CI 65.1–78.9%) for YD. Forest plots were generated to illustrate the results (Fig 1).

**Table 3:** Phase 1 comparative accuracy of the YD line probe assay versus Hain V1 line probe assay on characterized strains.

	RIF		INH		MDR	
	Sensitivity (95% CI)	Specificity (95% CI)	Sensitivity (95% CI)	Specificity (95% CI)	Sensitivity (95% CI)	Specificity (95% CI)
Hain V1	90.2% (84.8%, 94.2%) [157/174]	98.5% (95.7%, 99.7%) [198/201]	89.0% (83.8%, 93%) [178/200]	99.4% (96.9%, 100%) [174/175]	83.8% (77%, 89.2%) [129/154]	99.1% (96.8%, 99.9%) [219/221]
YD	72.4% (65.1%, 78.9%) [126/174]	98% (95%, 99.5%) [197/201]	92% (87.3%, 95.4%) [184/200]	92.6% (87.6%, 96%) [162/175]	71.4% (63.6%, 78.4%) [110/154]	97.3% (94.2%, 99%) [215/221]
Difference (YD–Hain V1)	-17.8% (-25.1%, -11.1%)	-0.5 (-3.4%, +2.2%)	+3% (+0.8%, +7.3%)	-6.9% (-11.8%, -3.2%)	-12.3% (-20.3%, -4.6%)	-1.8% (-5.0%, +0.9%)
Ni-margin	-3	-2	-10	-5	NA	NA

RIF, rifampicin; INH, isoniazid; MDR, multidrug-resistant TB. Accuracy of YD and Hain V1 compared to a composite reference standard is displayed followed by the comparative difference (YD-Hain V1) and non-inferiority margins. Each comparison has the point estimate followed by the 95% confidence interval in parenthesis, ‘()’. Brackets ‘[]’ show the number of successful test runs (compared to the reference standard) divided by the total number of tests. Ni-margin is the non-inferiority margin set a priori as we are not formally comparing non-inferiority for overall MDR, they is no corresponding MDR NI-margin (NA).

A per-probe analysis from results with the same set of strains was conducted using the composite reference standard to further investigate discrepant results between the LiPAs. The YD probes binding to the *oxyR-ahpC* intergenic region were not assessed in this sub-analysis, as targeted sequencing data was only available for *rpoB*, *katG* and *inhA*. Two strains were excluded, one containing a resistant/wild-type mix as determined by sequencing and the other with no sequencing data. Overall, 374 strains were analyzed between the two sites (Table 4). Pooled probe sensitivity (excluding the *oxyR-ahpC* gene region) was 70.5% (95%CI 66.1–74.7%) and specificity was 94.9% (95%CI 94.2–95.5%) for the detection of MDR-TB genotypes.

**Table 4:** Phase 1 per-probe performance analysis on characterized strains.

YD Probes	True Positives	False Negatives	False Positives	True Negatives	Sensitivity % (95%CI)	Specificity % (95%CI)
katG WT1	89	2	65	218	97.8% (92.3%, 99.7%)	77% (71.7%, 81.8%)
katG MT1	77	14	59	224	84.6% (75.5%, 91.3%)	79.2% (73.9%, 83.7%)
inhA 15 MT	13	15	5	341	46.4% (27.5%, 66.1%)	98.6% (96.7%, 99.5%)
inhA 8 MT	0	29	2	343	0% (0%, 11.9%)	99.4% (97.9%, 99.9%)
inhA 15 WT	25	3	13	333	89.3% (71.8%, 97.7%)	96.2% (93.7%, 98%)
inhA 8 WT	1	1	0	372	50% (1.3%, 98.7%)	100% (99%, 100%)
rpoB WT1	4	2	1	367	66.7% (22.3%, 95.7%)	99.7% (98.5%, 100%)
rpoB WT2	4	8	3	359	33.3% (9.9%, 65.1%)	99.2% (97.6%, 99.8%)
rpoB WT3	0	0	1	373	NA% (NA%, NA%)	99.7% (98.5%, 100%)
rpoB WT4	0	24	0	350	0% (0%, 14.2%)	100% (99%, 100%)
rpoB WT5	58	0	45	271	100% (93.8%, 100%)	85.8% (81.4%, 89.4%)
rpoB MT1	42	8	41	283	84% (70.9%, 92.8%)	87.3% (83.2%, 90.8%)
rpoB MT2	3	21	6	344	12.5% (2.7%, 32.4%)	98.3% (96.3%, 99.4%)
rpoB MT3	5	7	5	357	41.7% (15.2%, 72.3%)	98.6% (96.8%, 99.6%)
Pooled	321	134	246	4535	70.5% (66.1%, 74.7%)	94.9% (94.2%, 95.5%)

Per-probe analysis: comparison of YD results to phenotypic DST and targeted Sanger sequencing. Each probe for which there is corresponding sequencing data was analyzed separately. Pooled results appear in the last row. Note: *oxyR-ahpC* sequencing data was not available. However, *ahpC* wild type probes were absent for 40 strains. Twenty-seven were isoniazid-resistant by phenotypic drug susceptibility testing.

### Phase 2 results

A total of 488 sputum samples were tested by both LiPAs, with 440 samples included in the final diagnostic performance analysis (Table 5).

**Table 5:** Phase 2 sample characteristics and total number of samples included in the line probe assay comparative performance assessment by site.

	South Africa	Germany	Total
Total collected	214	274	488
NTM	4	0	4
S+/C-	9	5	14
No YD Results	10	15	25
Indeterminate YD results (RIF)	5	0	5
Indeterminate YD results (INH)	5	0	5
Total excluded	28	20	44
Total analyzed	186	254	440

Exclusions by site for sputum data. Note: Only 5 YD indeterminate samples are excluded in each comparison (e.g. INH, RIF) so the total excluded for South Africa is per comparison.

**Fig 2:**
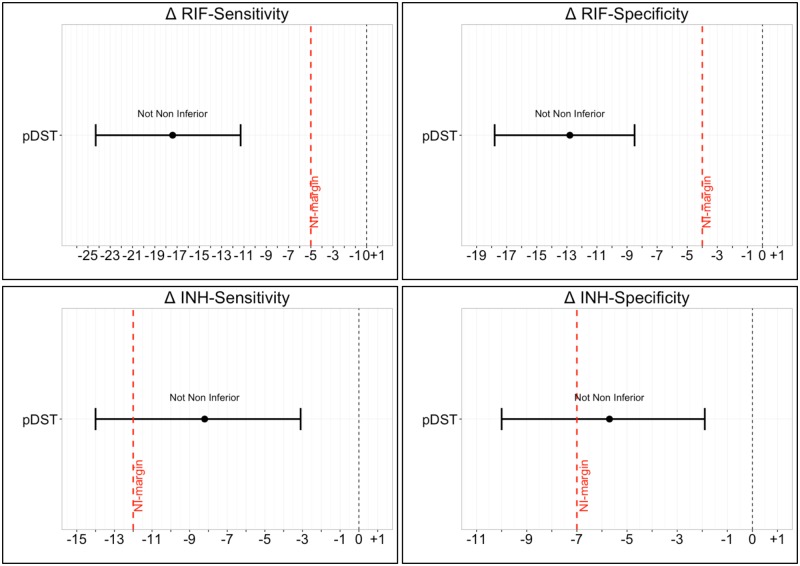
Phase 2 comparative accuracy of the YD line probe assay versus Hain V1 line probe assay on sputum samples. The difference in sensitivity/specificity (Δ = YD–Hain V1) is displayed in the CIs in each plot. The horizontal axis indicates the percent difference between tests. The point in the center of each CI represents the point estimate and whiskers representing the upper and lower limit of the 95% CIs. The black vertical dotted line (where visible) indicates zero difference in sensitivity/specificity and the red vertical broken line indicates the non-inferiority margin. Non-inferiority is demonstrated for a given comparison if the lower limit of the 95%CI does not cross the red broken line (non-inferiority margin).

Non-inferiority of the YD assay, compared to the Hain V1 assay, was not achieved for any accuracy parameter (Table 6). As in phase 1, the sensitivity of the assays for RIF resistance detection showed the largest discordance: 97.0% (95%CI 93.2–99%) for Hain V1 and 79.6% (95%CI 72.7–85.5%) for YD. Forest plots were generated to illustrate the results (Fig 2).

**Table 6:** Phase 2 comparative accuracy of the YD line probe assay versus Hain V1 line probe assay on sputa.

	RIF		INH		MDR	
	Sensitivity (95% CI)	Specificity (95% CI)	Sensitivity (95% CI)	Specificity (95% CI)	Sensitivity (95% CI)	Specificity (95% CI)
Hain V1	97% (93.2%, 99%) [162/167]	97.1% (94.3%, 98.7%) [265/273]	92.8% (88.2%, 96.0%) [180/194]	95.5% (92.1%, 97.7%) [235/246]	93.4% (88.2%, 96.8%) [142/152]	96.2% (88.2%, 96.8%) [276/287]
YD	79.6% (72.7%, 85.5%) [133/167]	84.2%(79.4%, 88.4%) [230/273]	84.5% (78.7%, 89.3%) [164/194]	89.8% (85.4%, 93%) [221/246]	75.7% (68.0%, 82.2%) [115/152]	92.0% (88.2%, 94.9%) [264/287]
Difference (YD–Hain V1)	-17.4% (-24.3%, -11.3%)	-12.8% (-17.8%, -8.5%)	-8.2% (-14.0%, -3.1%)	-5.7% (-10.0%, -1.9%)	-17.8% (-25.5%, +10.6%)	-4.2%(-8.0%, +0.8%)
Ni-margin	-5	-4	-12	-7	NA	NA

Accuracy of YD and Hain V1 compared to a phenotypic reference standard are displayed followed by the comparative difference (YD-Hain V1) and non-inferiority margins. Each comparison has the point estimate followed by the 95% confidence interval in parenthesis, ‘()’. Brackets ‘[]’ show the number of successful test runs (compared to the reference standard) divided by the total number of tests. Ni-margin is the non-inferiority margin set a priori as we are not formally comparing non-inferiority for overall MDR, they is no corresponding MDR NI-margin (NA).

## Discussion

The goal of this study was to directly compare the performance of YD to the WHO-endorsed Hain V1 assay for the detection of first-line drug resistance and MDR-TB. The YD LiPA did not achieve non-inferiority to the Hain V1 LiPA for the detection of MDR-TB in either characterized strains or sputum samples. Findings point to a need for a redesign of the YD LiPA to improve assay performance for first-line drug resistance detection in diverse sample types.

Notably, the sensitivity of the YD LiPA was much lower than the Hain V1 assay for the detection of RIF resistance in both characterized strains and clinical samples (72–80% compared to 90–97% for Hain V1). As the YD and Hain V1 assay probes cover the same *rpoB* gene regions for RIF resistance determination, the lower sensitivity seen for the YD assay likely reflects poor probe performance for certain mutations. Indeed, the per-probe performance assessment in phase 1 of this study found the YD *rpoB* mutation probes to perform with variable sensitivity. A range of hybridization issues were seen in this per-probe analysis, with both mutated and wildtype amplicons failing to hybridize to corresponding probes (e.g. the false negatives seen for YD *rpoB* probes WT4 and MT2) or hybridizing to incorrect probes (e.g. the false positives seen for YD *rpoB* probes WT5 and MT1). The large number of false negative and false positive results observed for these YD probes suggests that these probes should be re-evaluated, and possibly redesigned, to enhance their specificity for specific *rpoB* sequences and improve the overall sensitivity and specificity of the YD LiPA for RIF resistance detection.

For INH resistance detection, non-inferiority of YD was only achieved for the sensitivity of INH resistance detection using characterized strains in phase 1. Non-inferiority was not achieved for INH resistance detection in phase 2. Specifically, in phase 2, the sensitivity and specificity of the YD LiPA was lower than the Hain V1 assay (85% vs. 93%, and 90% vs. 96%, respectively), despite the inclusion of the *oxyR-ahpC* intergenic region in the YD LiPA. Although the YD LiPA assay did detect *oxyR-ahpC* intergenic region mutations in INH-resistant strains in this study (27 strains in phase 1 and 16 strains in phase 2), the detection of these mutations did not improve overall YD performance enough to achieve non-inferiority with Hain V1 for INH resistance or MDR-TB detection. This may be the result of these *ahpC* mutations co-occurring with resistance mutations in other genes (*katG* and/or *inhA*), as has been noted in previous studies [17–19], or, as with the *rpoB* probes, poor overall probe performance for the INH resistance mutations, especially for clinical samples (phase 2) where the bacillary burden may be lower compared to cultured strains (phase 1). It is also possible that the detection of *ahpC* mutations may have been interpreted as false positives against phenotypic DST results, if these mutations did not confer resistance above the set, INH critical concentration used in this study (0.1 μg/ml) [18]. Although the inclusion of the *ahpC* gene region may ultimately decrease the number of false positives and improve the performance if LiPAs for INH resistance and MDR-TB detection, this cannot be confirmed without additional studies and detailed sequencing data.

The study had several important strengths. First, the Hain V1 and YD assays were performed on the same DNA suspension, ensuring that neither LiPA was performed on a higher-quality sample. Second, the study included a South African patient population, where *ahpC* mutations have been previously reported to occur at high frequencies [17, 20]. Third, the use of a composite reference standard that included genotypic data during phase 1 minimized bias and allowed for additional insights on test performance. Finally, this study highlights the importance of validating novel MDR-TB molecular diagnostic performance in independent studies. Although the initial performance estimates of a new diagnostic may appear promising in local assessments, an external evaluation might reveal significant limitations to the technology that would have dire consequences for TB and DR-TB control efforts in high burden regions if these platforms were to be directly implemented in sites of intended use. A well-controlled, independent evaluation can identify these failings and pinpoint specific areas for assay improvement prior to further assessment.

Study limitations include the fact that sequencing was not done on the phase 2 clinical samples, which could have resulted in some misclassifications of strains that carry phenotypically sensitive mutations associated with resistance [21]. Furthermore, since the sequencing of characterized strains was done prior to this study, the *oxyR-ahpC* intergenic region was not sequenced, limiting our ability to confirm the additional contribution of the *ahpC* mutations to the sensitivity of the YD LiPA for INH resistance. However, in this study, the *ahpC* promoter mutations that were detected by the YD assay did not appear to increase assay performance for INH resistance and MDR detection enough to demonstrate non-inferiority with Hain V1. Additionally, the Hain V1 assay had been used at the study sites prior to this study, potentially putting the YD assay at a possible disadvantage. Nonetheless, both sites were reference laboratories experienced in carrying out complex tests, and the diagnostic developers and site staff considered the training received prior to the study to be sufficient to assess non-inferiority of the two LiPAs.

Although molecular diagnostics with expanded gene coverage for TB resistance-detection promise higher sensitivity for MDR-TB detection, it is critical to evaluate their performance against the performance of similar, WHO-endorsed technologies for diverse sample types. In this study, the YD LiPA did not achieve non-inferiority against the Hain V1 LiPA for MDR-TB detection in either characterized strains or clinical samples. While the inclusion of the *oxyR-ahpC* intergenic region may increase the performance of LiPAs for INH resistance detection in certain populations, the YD assay had notable performance lapses, likely due to lack of probe specificity. Future research should focus on ways to improve the accuracy of the YD LiPA, such as redesigning the probes, prior to re-evaluating the performance of the assay to determine its utility for MDR-TB diagnosis.

## Supporting information

S1 Text Phase 1 nontuberculous mycobacteria. (DOCX)

S1 Table A composite reference standard was derived from sequencing and DST. (DOCX)

S2 Table Phase 1 comparative accuracy of the Hain V2 line probe assay versus Hain V1 line probe assay on characterized strains. (DOCX)

S3 Table Phase 2 comparative accuracy of the Hain V2 line probe assay versus Hain V1 line probe assay on sputa. (DOCX)

S1 Fig Phase 1 comparative accuracy of the Hain V2 line probe assay versus Hain V1 line probe assay on characterized strains. (DOCX)

S2 Fig Phase 2 comparative accuracy of the Hain V2 assay versus Hain V1 line probe assay on sputum samples. (DOCX)

S1 Dataset Full dataset for all strains included in the phase 1 study. (CSV)

S2 Dataset Strains included in the phase 1 study analyses. (CSV)

S3 Dataset Full dataset for all sputa include in the phase 2 study. (CSV)

S4 Dataset Sputa included in the phase 2 study analyses. (CSV)
