# Supplementary material for: Comparative accuracy of the REBA MTB MDR and Hain MTBDRplus line probe assays for the detection of multidrug-resistant tuberculosis: A multicenter, non-inferiority study
Source: PLoS One. 2017 Mar 24;12(3):e0173804. doi: 10.1371/journal.pone.0173804 (PMC5365104; doi:10.1371/journal.pone.0173804)
Supplement: S1 Text — (DOCX) [file pone.0173804.s001.docx]

**S1 Text. Phase 1 nontuberculous mycobacteria.**

In phase 1, 21 NTM were tested between the two sites. One NTM was indeterminate by YD assay. Of the remaining 20 strains tested: eight were called NTM by YD (mycobacteria probe present, MTBC probe absent); ten were called MTBC by YD (both probes present); and two were called non-mycobacteria (both probes absent).
