# Supplementary material for: Comparative accuracy of the REBA MTB MDR and Hain MTBDRplus line probe assays for the detection of multidrug-resistant tuberculosis: A multicenter, non-inferiority study
Source: PLoS One. 2017 Mar 24;12(3):e0173804. doi: 10.1371/journal.pone.0173804 (PMC5365104; doi:10.1371/journal.pone.0173804)
Supplement: S1 Table — (DOCX) [file pone.0173804.s002.docx]

**S1 Table. A composite reference standard was derived from sequencing and DST.**

| **DST Result** | **Sequencing Result** | **Composite (cDST) Result** |
| --- | --- | --- |
| Resistant | Mutations associated with resistance | Resistant |
| Sensitive | Mutations associated with resistance | Resistant |
| Sensitive | No mutations or mutations not associated with resistance | Sensitive |
| Resistant | No mutations or mutations not associated with resistance | Resistant |
