# Supplementary material for: Comparative accuracy of the REBA MTB MDR and Hain MTBDRplus line probe assays for the detection of multidrug-resistant tuberculosis: A multicenter, non-inferiority study
Source: PLoS One. 2017 Mar 24;12(3):e0173804. doi: 10.1371/journal.pone.0173804 (PMC5365104; doi:10.1371/journal.pone.0173804)
Supplement: S2 Table — (DOCX) [file pone.0173804.s003.docx]

**S2 Table. Phase 1 comparative accuracy of the Hain V2 line probe assay versus Hain V1 line probe assay on characterized strains.**

|  | RIF | | INH | | | MDR | |
| --- | --- | --- | --- | --- | --- | --- | --- |
|  | Sensitivity  (95% CI) | Specificity  (95% CI) | Sensitivity  (95% CI) | Specificity  (95% CI) | | Sensitivity  (95% CI) | Specificity  (95% CI) |
| Hain V1 | 90.3%  (84.9%, 94.2%)  [158/175] | 98.5%  (95.7%, 99.7%)  [196/199] | 89.1%  (83.9%, 93.0%)  [179/201] | 99.4%  (96.9%, 100.0%)  [174/175] | | 83.9%  (77.1%, 89.3v)  [130/155] | 99.1%  (96.7%, 99.9%)  [217/219] |
| Hain V2 | 90.3%  (84.9%, 94.2%)  [158/175] | 98.5%  (95.7%, 99.7%)  [196/199] | 89.1%  (83.9%, 93.0%)  [179/201] | 99.4%  (96.9%, 100.0%)  [174/175] | | 83.9%  (77.1%, 89.3%)  [130/155] | 99.1%  (96.7%, 99.9%)  [217/219] |
| Difference  ( Hain V2 – Hain V1) | 0.0%  (-2.1%, 2.1%) | 0.0%  (-1.9%, 1.9%) | 0.0%  (-1.9%, 1.9%) | 0.0%  (-2.1%, 2.1%) | | 0.0%  (-2.4%, 2.4%) | 0.0%  (-1.7%, 1.7%) |
| Ni-margin | -3 | -2 | -10 | | -5 | NA | NA |

Accuracy of Hain V2 and Hain V1 compared to a phenotypic reference standard are displayed followed by the comparative difference (YD-Hain V1) and non-inferiority margins. Each comparison has the point estimate followed by the 95% confidence interval in parenthesis, ‘()’. Brackets ‘[]’ show the number of successful test runs (compared to the reference standard) divided by the total number of tests. Ni-margin is the non-inferiority margin set a priori as we are not formally comparing non-inferiority for overall MDR, they is no corresponding MDR NI-margin (NA).
