# Supplementary material for: Comparative accuracy of the REBA MTB MDR and Hain MTBDRplus line probe assays for the detection of multidrug-resistant tuberculosis: A multicenter, non-inferiority study
Source: PLoS One. 2017 Mar 24;12(3):e0173804. doi: 10.1371/journal.pone.0173804 (PMC5365104; doi:10.1371/journal.pone.0173804)
Supplement: S3 Table — (DOCX) [file pone.0173804.s004.docx]

**S3 Table. Phase 2 comparative accuracy of the Hain V2 line probe assay versus Hain V1 line probe assay on sputa.**

|  | RIF | | INH | | MDR | |
| --- | --- | --- | --- | --- | --- | --- |
|  | Sensitivity  (95% CI) | Specificity  (95% CI) | Sensitivity  (95% CI) | Specificity  (95% CI) | Sensitivity  (95% CI) | Specificity  (95% CI) |
| Hain V1 | 97.1%  (93.3%, 99.0%)  [166/171] | 97.1%  (94.3%, 98.7%)  [267/275] | 94.4%  (90.2%, 97. 2%)  [186/197] | 96.4%  (93.2%, 98.3%)  [240/249] | 94.7%  (89.9%, 97.7%)  [144/152] | 96.9%  (94.1%, 98.6%)  [278/287] |
| Hain V2 | 98.2%  (95.0%, 99.6%)  [168/171] | 97.8%  (95.3%, 99.2%)  [269/275] | 95.4%  (91.5%, 97.9%)  [188/197] | 98.8%  (96.5%, 99.8%)  [246/249] | 96.7%  (92.5%, 98.9%)  [147/152] | 98.3%  (96.0%, 99.4%)  [282/287] |
| Difference  ( Hain V2 – Hain V1) | 1.2%  (-1.1%, 4.2%) | 0.7%  (-1.0%, 2.8%) | 1.0%  (-1.5%, 3.9%) | 2.4%  (0.2%, 5.3%) | 2.0%  (-0.5%, 5.6%) | 0.4%  (0.05%, 3.5%) |
| Ni-margin | -5 | -4 | -12 | -7 | NA | NA |

Accuracy of Hain V2 and Hain V1 compared to a phenotypic reference standard are displayed followed by the comparative difference (YD-Hain V1) and non-inferiority margins. Each comparison has the point estimate followed by the 95% confidence interval in parenthesis, ‘()’. Brackets ‘[]’ show the number of successful test runs (compared to the reference standard) divided by the total number of tests. Ni-margin is the non-inferiority margin set a priori as we are not formally comparing non-inferiority for overall MDR, they is no corresponding MDR NI-margin (NA).
