# Supplementary material for: Comparative accuracy of the REBA MTB MDR and Hain MTBDRplus line probe assays for the detection of multidrug-resistant tuberculosis: A multicenter, non-inferiority study
Source: PLoS One. 2017 Mar 24;12(3):e0173804. doi: 10.1371/journal.pone.0173804 (PMC5365104; doi:10.1371/journal.pone.0173804)
Supplement: S2 Fig — (DOCX) [file pone.0173804.s006.docx]

**S2 Fig.** **Phase 2 comparative accuracy of the Hain V2 assay versus Hain V1 line probe assay on sputum samples.**


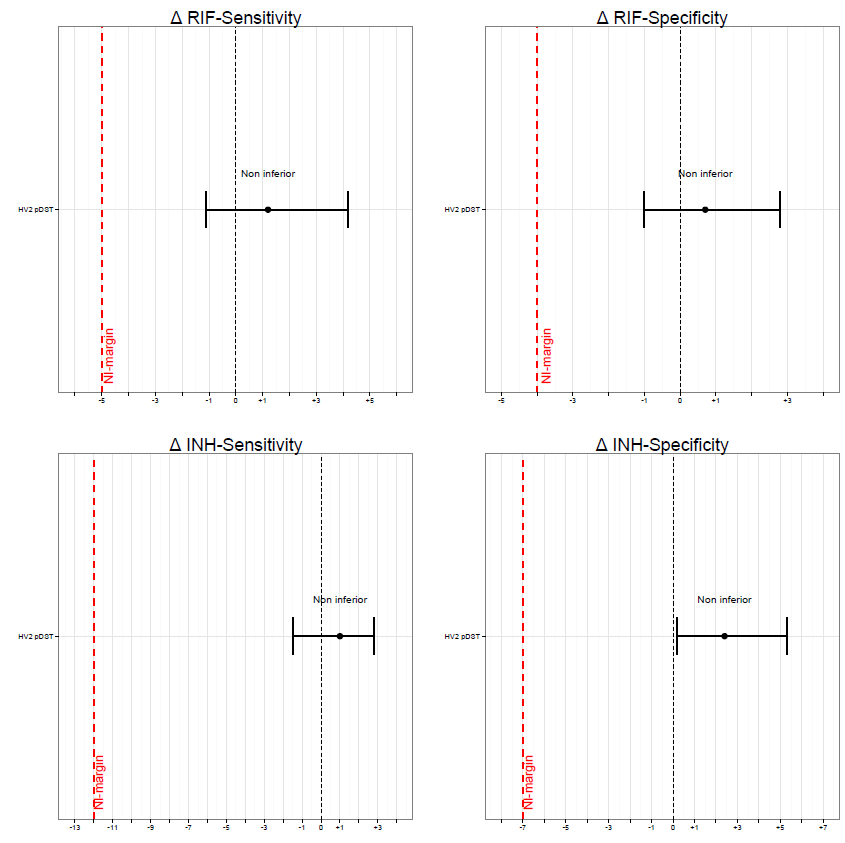


The difference in sensitivity/specificity (∆ = Hain V2 – Hain V1) is displayed in the CIs in each plot. The horizontal axis indicates the percentage difference between tests. The point in the center of each CI represents the point estimate and whiskers representing the upper and lower limit of the 95% CIs. The black vertical dotted line (where visible) indicates zero difference in sensitivity/specificity and the red vertical broken line indicates the non-inferiority margin. Non-inferiority is demonstrated for a given comparison if the lower limit of the 95%CI does not cross the red broken line (non-inferiority margin).
